# Restoring Ampicillin Sensitivity in Multidrug-Resistant *Escherichia coli* Following Treatment in Combination with Coffee Pulp Extracts

**DOI:** 10.4014/jmb.2304.04051

**Published:** 2023-05-24

**Authors:** Anchalee Rawangkan, Atchariya Yosboonruang, Anong Kiddee, Achiraya Siriphap, Grissana Pook-In, Ratsada Praphasawat, Surasak Saokaew, Acharaporn Duangjai

**Affiliations:** 1 Division of Microbiology and Parasitology, School of Medical Sciences, University of Phayao, Phayao 56000, Thailand; 2 Unit of Excellence in Research and Product Development of Coffee, Division of Physiology, School of Medical Sciences, University of Phayao, Phayao 56000, Thailand; 3 Department of Pathology, School of Medicine, University of Phayao, Phayao 56000, Thailand; 4 Division of Social and Administrative Pharmacy, Department of Pharmaceutical Care, School of Pharmaceutical Sciences, University of Phayao, Phayao 56000, Thailand; 5 Centre of Health Outcomes Research and Therapeutic Safety (Cohorts), School of Pharmaceutical Sciences, University of Phayao, Phayao 56000, Thailand; 6 Unit of Excellence on Clinical Outcomes Research and Integration (UNICORN), School of Pharmaceutical Sciences, University of Phayao, Phayao 56000, Thailand; 7 Division of Physiology, School of Medical Sciences, University of Phayao, Phayao 56000, Thailand

**Keywords:** Antimicrobial activity, ampicillin, coffee pulp, combination, *Escherichia coli*, multidrug-resistant

## Abstract

*Escherichia coli*, particularly multidrug-resistant (MDR) strains, is a serious cause of healthcare-associated infections. Development of novel antimicrobial agents or restoration of drug efficiency is required to treat MDR bacteria, and the use of natural products to solve this problem is promising. We investigated the antimicrobial activity of dried green coffee (DGC) beans, coffee pulp (CP), and arabica leaf (AL) crude extracts against 28 isolated MDR *E. coli* strains and restoration of ampicillin (AMP) efficiency with a combination test. DGC, CP, and AL extracts were effective against all 28 strains, with a minimum inhibitory concentration (MIC) of 12.5–50 mg/ml and minimum bactericidal concentration of 25–100 mg/ml. The CP–AMP combination was more effective than CP or AMP alone, with a fractional inhibitory concentration index value of 0.01. In the combination, the MIC of CP was 0.2 mg/ml (compared to 25 mg/ml of CP alone) and that of AMP was 0.1 mg/ml (compared to 50 mg/ml of AMP alone), or a 125-fold and 500-fold reduction, respectively, against 13-drug resistant MDR *E. coli* strains. Time-kill kinetics showed that the bactericidal effect of the CP–AMP combination occurred within 3 h through disruption of membrane permeability and biofilm eradication, as verified by scanning electron microscopy. This is the first report indicating that CP–AMP combination therapy could be employed to treat MDR *E. coli* by repurposing AMP.

## Introduction

Nosocomial infections caused by antimicrobial-resistant pathogens are a leading cause of death worldwide. *Escherichia coli* is the most common pathogen responsible for resistance-related fatalities, followed by *Staphylococcus aureus*, *Klebsiella pneumoniae*, *Streptococcus pneumoniae*, *Acinetobacter baumannii*, and *Pseudomonas aeruginosa* [1]. In hospitalized patients, *E. coli* is a common cause of healthcare-associated infections, which cause pneumonia, urinary tract infections, and bloodstream infections [2]. Some strains of *E. coli* have become very resistant to several antibiotics and are, therefore, described as multidrug-resistant (MDR) *E. coli*. In recent years, new drugs, *i.e.*, ceftolozane/tazobactam, ceftazidime/avibactam, meropenem/vaborbactam, imipenem/cilistatin/relebactam, cefiderocol, plazomicin, eravacycline, and omadacycline, have been used to treat gram-negative MDR bacterial infections [3, 4]. However, their long-term effectiveness should be ensured in order to postpone as much as possible the emergence and spread of resistance to novel agents.

Since ampicillin (AMP), a semi-synthetic beta-lactam antibiotic that inhibits the synthesis of the bacterial cell wall, has been widely used to treat *E. coli* infections for many years, the rate of resistance against it has increased; consequently, it is not recommended for treating *E. coli* infections at present. The common mechanisms of bacterial resistance against antibiotics are encoding of beta-lactamase, modification of the target protein in the cell wall, reduction of exterior membrane permeability, and boosting of drug efflux pump expression [5]. Recently, AMP was repurposed by combining it with another antibiotic agent, such as the AMP–ceftriaxone combination treatment for *Enterococcus faecalis* infection, as demonstrated in in vitro, in vivo, and case report studies [6-8], and AMP–azithromycin combination treatment for *S. pneumoniae* infection, as demonstrated in in vitro, in vivo, and patient studies [9, 10]. Therefore, the combination of AMP and another compound might serve as an alternative for treating MDR *E. coli*.

Coffee (*Coffea* L.) is one of the most popular beverages globally, especially the *C. arabica* L. cv. caturra (arabica) cultivar [11]. During coffee cultivation and preparation, a large amount of by-products and waste is produced, *i.e.*, spent coffee grounds, coffee husks, leaf, peel, and pulp [12]. We recently found that coffee beans and coffee by-product extracts, which are high in phenolic compounds and antioxidant activity, have a wide range of health benefits as well as antibacterial activity against gram-positive and -negative food-borne pathogens, including *Vibrio cholerae*, *S. aureus*, *Bacillus subtilis*, *E. coli*, and *Salmonella typhimurium* [13-16]. Therefore, an extract from the coffee plant could be applied as an alternative treatment for an assortment of bacterial infections.

In this study, we investigated the efficiency of dried green coffee (DGC) beans and coffee by-products, including coffee pulp (CP) and arabica leaf (AL) crude extracts, against the antibacterial properties of MDR *E. coli* strains and the combination of these extracts with AMP, as well as the mechanism of action.

## Materials and Methods

### Preparation of Coffee Beans and Coffee by-Products

Coffee beans and coffee by-products (voucher number NU003806) were kindly provided by the Chao-Thai-Pukao Factory, Chiang Mai Province, Thailand. The crude extract powders of DGC, CP, and AL were prepared by boiling in hot water and lyophilizing, as described in our previous reports [14, 16].

### Bacterial Strains

Twenty-eight MDR *E. coli* strains were recovered from houseflies in Phayao Hospital, Phayao Province, Thailand, as described previously [17]. All strains were tested with 15 antibiotics: ampicillin (AMP), amoxycillin (AML), cephalotin (KF), cefotaxime (CTX), chloramphenicol (C), trimethoprim-sulfamethoxazole (SXT), meropenem (MEM), imipenem (IMP), amikacin (AK), gentamicin (CN), ciprofloxacin (CIP), norfloxacin (NOR), amoxicillin/clavulanic acid (AMC), ampicillin/sulbactam (SAM), and tetracycline (TE). Table S1 shows the profiles of antibacterial resistance of MDR *E. coli* strains to the 15 antimicrobial agents. The typical reference strain was *E. coli* ATCC 25922.

### Minimum Inhibitory Concentration and Minimum Bactericidal Concentration Assay

The sensitivity of the extract to the MDR *E. coli* strains was determined using the microdilution method, as recommended by the Clinical and Laboratory Standards Institute (CLSI) [18]. The DGC, CP, and AL preparations were serially diluted in Mueller–Hinton broth to create a concentration range from 0.05 mg/ml to 100 mg/ml. Each bacterial culture was added at 5 × 10^5^ CFU/ml and incubated for 24 h at 37°C. Resazurin was then used to clarify the minimum inhibitory concentration (MIC). The minimum bactericidal concentration (MBC) was determined when there was no colony growth on Mueller–Hinton agar by the drop test, as described in previous studies [14, 16]. AMP, which the CDC no longer recommends as a first-line agent to treat *E. coli* infections, was also tested.

### Checkerboard Assays

To investigate the potential synergistic effects of DGC, CP, and AL extracts when combined with AMP, checkerboard assays were carried out with the MDR *E. coli* E48 representative strain, following previous reports [14, 16, 19]. The fractional inhibitory concentration index (FICI) was calculated using the formula: FIC (a) = MIC of extracts in the combination/MIC of extracts alone; FIC (b) = MIC of AMP in the combination/MIC of AMP alone; FICI = FIC (a) + FIC (b). A synergistic effect is indicated for an FICI of < 0.5, an additive effect for an FICI of 0.5–4, and an antagonistic effect for an FICI > 4 [20, 21].

### Time-Kill Kinetics Assay

The time-kill assay was used to investigate the pharmacokinetics of the coffee extract alone and in combination with AMP. Various concentrations of the extract, *i.e.*, 1×, 2×, and 4× MIC, 1× MIC of AMP, and the combination of the extract and AMP, were used to determine bacterial cell growth at 0, 1, 2, 4, 8, 16, and 24 h. The kill curve defined the bactericidal effect as a reduction of ≥ 3 log10 CFU/ml relative to the initial inoculum (5 × 10^5^ CFU/ml), whereas the bacteriostatic effect corresponded to a < 3 log10 CFU/ml decrease relative to the initial inoculum, as previously described [14, 16, 22].

### Outer Membrane Disruption Analysis

Firstly, we determined the leakage of DNA and proteins from the cell membrane. The MDR *E. coli* E48 strain was treated with the appropriate concentrations of the extract, AMP, and extract–AMP combination for 1 h at 37°C. The amount of DNA was measured as optical density (OD) at 260 nm using a NANO-400A Micro Spectrophotometer (Hangzhou Allsheng Instruments Co., Ltd., China), and the amount of protein was measured using a Bio-Rad DC Protein Assay Kit (Bio-Rad Laboratories, Inc., USA). Next, the degree of outer membrane disruption was determined by cell staining with N-phenyl-1-naphthylamine (NPN) and rhodamine 123 (Rh123) fluorescence dyes. The fluorescence intensity of NPN (OD at 350/420 nm) and Rh123 (OD at 480/530 nm) were calculated as relative fluorescence intensity (%) = [F1/F0] ×100, where F0 was the fluorescence intensity of non-treated cells and F1 was the fluorescence intensity of treated cells. As a positive control, 0.1% Triton X-100 was used, as previously described [14, 16, 23].

### Biofilm Formation Assay

The biofilm formation assay was performed in accordance with previous studies [24-26], with some modifications. Briefly, the MDR *E. coli* E48 strain was inoculated at 1 × 10^8^ CFU/ml in a 96-well microtiter plate at a final volume of 200 μl with or without the extract alone (1×, 2×, and 4× MIC), AMP alone, or the combination of extract and AMP. After 24 h of incubation, nonadherent cells were removed by gentle washing with PBS and heat-dried at 60°C. Biofilm formation was determined by staining with 200 μl of 0.1% crystal violet for 15 min, after which the samples were washed with PBS twice. Crystal violet was dissolved in 200 μl of 95% ethanol for 20 min at room temperature and OD measured at 595 nm. The percentage of biofilm mass was calculated using the formula,[At/Ac] × 100, where Ac was OD595 for control wells and At was OD595 in the presence of a tested compound.

### Preformed Biofilm Biomass and Viability Assay

The biofilms of the MDR *E. coli* E48 strain were produced for 24 h, as described above. The planktonic cells were then discarded and the biofilm washed with PBS. The relevant treatment was added at a final volume of 200 μl and incubated for 24 h at 37°C. Biofilm biomasses were then assessed by 0.1% crystal violet staining. Preformed biofilm viability was assessed by staining with 5 mg/ml of 3-(4,5-dimethylthiazol-2-yl)-2,5-diphenyl tetrazolium bromide (MTT) for 30 min at 37°C. After discarding the solution, DMSO was added to the well and incubated for 30 min; then, the OD was determined at 570 nm [27].

### Scanning Electron Microscopy

The MDR *E. coli* E48 strain at 1 × 10^8^ CFU/ml was treated with 1× MIC of each extract and AMP, as well as the combination of the extract and AMP, at 37°C for 3 h. Bacterial cells were then harvested by centrifuging at 3,000 ×*g* for 5 min. We then resuspended the pellet in 1 ml of 2.5% glutaraldehyde and incubated it for 3 h at 4°C. After washing with PBS, the bacterial cells were dehydrated with an ethanol grade series (30%, 50%, 70%, and 90%) for 30 min per step and subjected to scanning electron microscopy (SEM; Tescan, Vega III, Czech Republic), according to previous reports [14, 16].

### Statistical Analysis

All analyses were performed by at least three independent laboratories. Data are reported as mean ± SD. A one-way ANOVA with Dunnett's multiple comparison test was performed in GraphPad Prism 5.01 (GraphPad Software, Inc., USA). The statistical significance was set at *p* < 0.05.

## Results

### Coffee Beans and Coffee by-Products Inhibit Multidrug-Resistant *E. coli* Strains

We first investigated the MICs and MBCs of extracts of coffee beans and coffee by-products, *i.e.*, DGC, CP, and AL extracts, against 28 MDR *E. coli* strains that maintained resistance to AMP, AML, KF, CTX, C, SXT, MEM, IMP, AK, CN, CIP, NOR, AMC, SAM, and TE. MDR *E. coli* strains were resistant to 4–13 of the 15 drugs tested. Table 1 shows that all 28 isolates were sensitized to DGC, CP, and AL extracts, with MICs ranging from 12.5 to 50 mg/ml and MBCs ranging from 25 to 100 mg/ml. The MIC of DGC against all 28 strains of MDR *E. coli* was 50 mg/ml, while MICs of CP were 12.5 mg/ml against 28.57% (8/28) of the strains, 25 mg/ml against 64.29% (18/28), and 50 mg/ml against 7.14% (2/28). The MICs of AL were 12.5 mg/ml against 14.29% (4/28) of the strains and 50 mg/ml against 85.71% (24/28). Against the *E. coli* ATCC 25922 reference strain, the MICs of DGC, CP, and AL were 50 mg/ml, 25 mg/ml, and 50 mg/ml, respectively. This suggests that DGC, CP, and AL inhibited MDR *E. coli* strains, and that CP might have affected MDR *E. coli* strains more than DGC and AL.

### The Combination of Coffee Pulp and Ampicillin Enhanced Antimicrobial Activity against Multidrug-Resistant *E. coli*

We next investigated the synergistic effects of DGC, CP, and AL extracts in a combination treatment with AMP against the MDR *E. coli* E48 strain using checkerboard assays. It is important to note that the MDR *E. coli* E48 strain shows the highest antibiotic resistance: it is resistant to 13 drugs (AMP, AML, KF, CTX, SXT, MEM, IPM, CN, CIP, NOR, AMC, SAM, and TE). Therefore, the E48 strain was chosen as a representative strain of MDR *E. coli* for future study. Moreover, the standard bioactive compounds in coffee, such as caffeine, chlorogenic acid, and caffeic acid, were also investigated. Table 2 shows the susceptibility of the MDR *E. coli* E48 strain to the extracts of DGC, CP, AL, standard coffee bioactive compounds, and AMP. The MIC of CP was 25 mg/ml, while that of DGC and AL was 50 mg/ml. Caffeine had the highest bioactive effect on MDR *E. coli*, with an MIC of 6.25 mg/ml, followed by chlorogenic acid (MIC = 12.25 mg/ml) and caffeic acid (MIC = 25 mg/ml).

In combination tests (Table 3), a synergistic outcome was observed only for the CP–AMP combination treatment with an FICI value of 0.01. The MIC of CP alone was 25 mg/ml, while that of the combination was 0.2 mg/ml, a 125-fold reduction. The MIC of AMP alone was 50 mg/ml, while that of the combination was 0.1 mg/ml, a 500-fold reduction. These findings suggest that the CP–AMP combination could be a more effective treatment for MDR *E. coli* than either CP or AMP alone.

### Analysis of the Bactericidal Kinetics of the Coffee Pulp–Ampicillin Combination Treatment

**Fig. 1:**
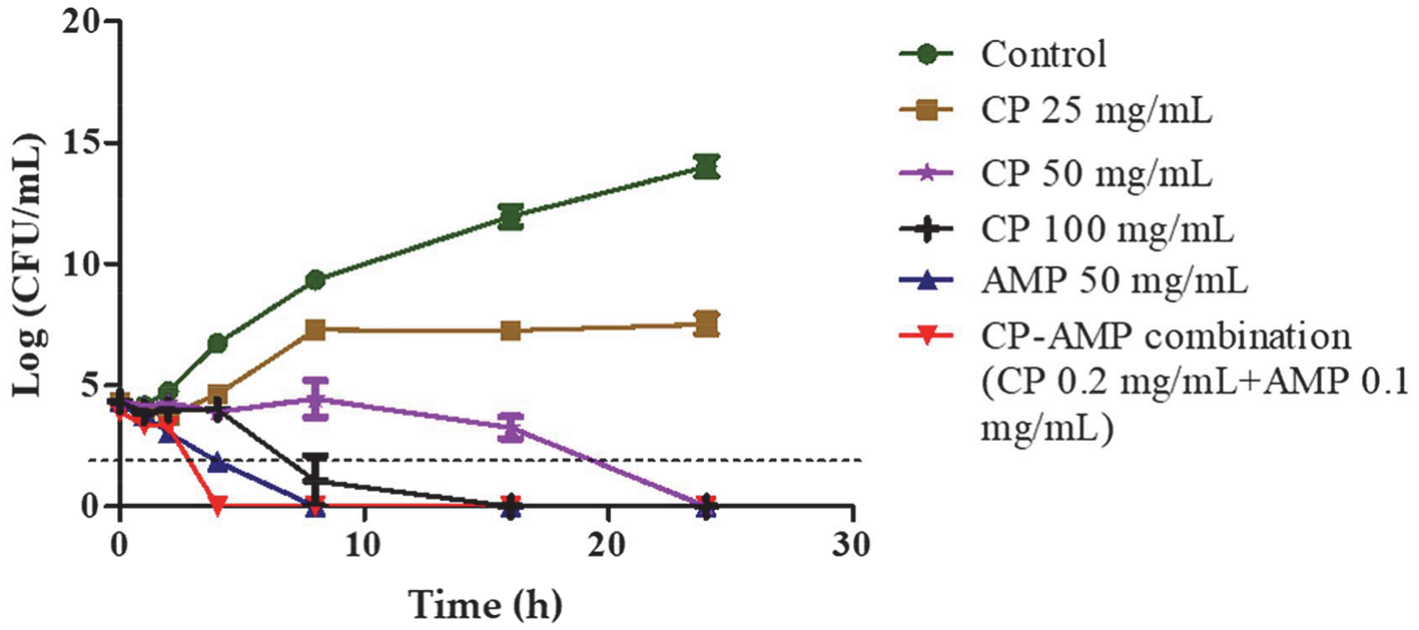
Effects of the coffee pulp–ampicillin (CP–AMP) combination against the multidrug-resistant *E. coli* E48 strain. The time-kill kinetics of the CP extract, AMP, and the CP–AMP combination were investigated. Bacterial samples were collected at 1, 2, 4, 8, 16, and 24 h to determine the number of viable bacteria. The dashed bars represent the bactericidal level.

To explore the pharmacological activity of the CP–AMP combination treatment, we investigated the effects of the bactericidal level in the kinetic growth curves of CP alone at various concentrations (25, 50, and 100 mg/ml), AMP alone (50 mg/ml), and the CP–AMP combination (CP 0.2 mg/ml + AMP 0.1 mg/ml) on the viability of the MDR *E. coli* E48 strain. Fig. 1 shows that CP suppressed bacterial cell growth in a dose-dependent manner, while the bacteria performed well in terms of exponential growth in a control group, increasing to approximately 14 log units within 24 h. CP at concentrations of 50 mg/ml and 100 mg/ml showed bactericidal activity within 20 h and 7 h, respectively. However, at a concentration of 25 mg/ml, it only had a bacteriostatic effect. Interestingly, the CP–AMP combination treatment exhibited bactericidal property within 3 h, whereas AMP demonstrated bactericidal property within 4 h after treatment, a reduction of 1 h. All these results clearly show that the CP–AMP combination was bactericidal against the tested MDR *E. coli* strains.

### Coffee Pulp–Ampicillin Combination Treatment Disrupts Membrane Permeability of Multidrug-Resistant *E. coli*

**Fig. 2:**
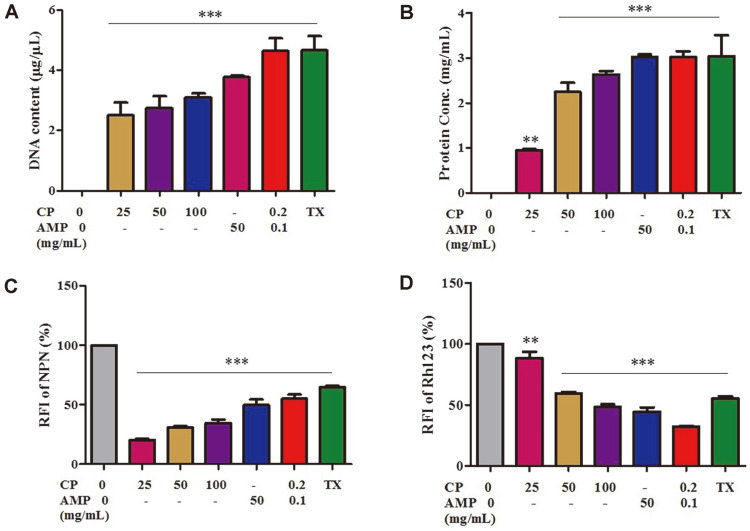
Effects of the coffee pulp–ampicillin (CP–AMP) combination on membrane permeability. The *E. coli* E48 strain was treated with CP alone (25, 50, 100 mg/ml), AMP alone (50 mg/ml), or the CP-AMP combination (CP 0.2 mg/ml + AMP 0.1 mg/ml) for 1 h at 37°C. The amounts of DNA (**A**) and proteins (**B**) were determined. The relative fluorescence intensity (RFI) of NPN (**C**) and Rh123 (**D**) was measured. The positive control was 0.1% Triton X-100 (TX). **p < 0.01, ***p < 0.001.

Normally, the effectiveness of the drug permeability barrier of the gram-negative cell wall is correlated with the permeability of the bacterial cell membrane. Therefore, we measured nucleotide and protein leakage and investigated membrane permeability using assays incorporating NPN and Rh123. As predicted, after 1 h of treatment with CP alone at various concentrations, DNA and protein leakage was induced in a dose-dependent manner. Furthermore, treatment with the CP–AMP combination caused DNA to be released from the cell at a greater rate than treatment with either CP or AMP alone, showing a strong effect of membrane permeability similar to that of Triton X-100 [28] (Fig. 2A). Similar results were seen for proteins (Fig. 2B). Treatment with the CP–AMP combination also altered membrane permeabilization by increasing the relative fluorescence intensity (RFI) of NPN and reducing the RFI of Rh123 more than treatment with CP or AMP alone (Figs. 2C and 2D). It should be emphasized that CP had a dose-dependent effect on outer membrane permeabilization. These findings indicate that the CP–AMP combination altered membrane potential activity and increased membrane permeability, resulting in leakage of intracellular contents and cell death.

### Coffee Pulp–Ampicillin Combination Treatment Inhibits Biofilm Formation and Reduces Preformed Biofilm of Multidrug-Resistant *E. coli*

**Fig. 3:**
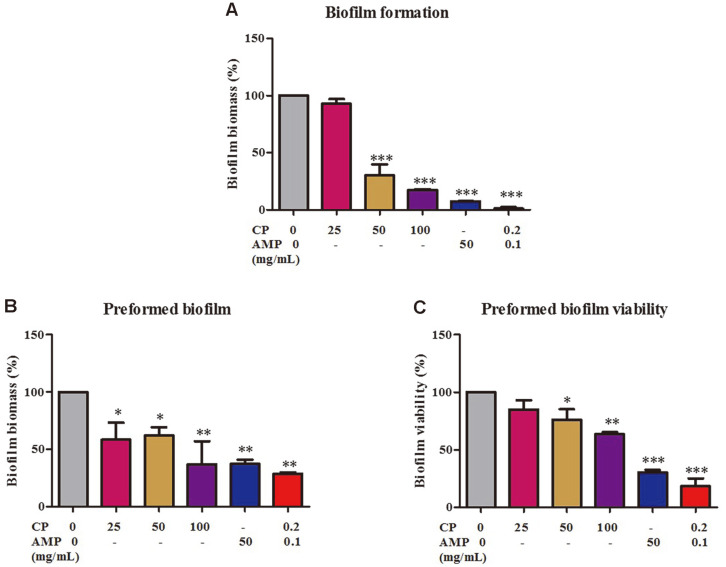
The effects of the coffee pulp–ampicillin combination on biofilm formation. The *E. coli* E48 strain was treated with CP alone (25, 50, 100 mg/ml), AMP alone (50 mg/ml), or the CP-AMP combination (CP 0.2 mg/ml+AMP 0.1 mg/ml) for the biofilm formation assay and the preformed biofilm biomass and viability assay. Biofilm formation (**A**) and preformed biofilm (**B**) were quantified using crystal violet staining. Preformed biofilm viability (**C**) was quantified with the MTT assay. *p < 0.05 **p < 0.01, ***p < 0.001.

Biofilms of *E. coli* are known to be highly resistant to the action of antibiotics. Therefore, we investigated the effects of the CP–AMP combination on disruption or elimination of biofilm. MDR *E. coli* E48 cells were treated with CP extract alone at concentrations of 25, 50, and 100 mg/ml (1×, 2×, and 4× MIC, respectively), or 50 mg/ml of AMP alone, or the combination of 0.2 mg/ml of CP and 0.1 mg/ml of AMP. Fig. 3A shows that CP inhibited biofilm formation in a dose-dependent manner, with a percentage biomass inhibition of 92.80 ± 4.29%, 30.19 ± 9.54%, and 17.27 ± 0.73% when treated with 25, 50, and 100 mg/ml, respectively, compared to the non-treated cells. On the other hand, treatment with 50 mg/ml of AMP inhibited biofilm biomass by up to 7.25 ± 4.29%. It is important to note that the CP–AMP combination inhibited biofilm biomass almost completely—a 99%reduction.

We also investigated the effect of the CP–AMP combination on biofilm elimination. One-day-old biofilms were exposed to CP extract alone, AMP alone, or the CP–AMP combination for 24 h. Biofilm biomass and biofilm viability were measured with crystal violet staining and MTT assay, respectively. CP treatment at concentrations of 25, 50, and 100 mg/ml decreased biofilm biomass by 58.5 ± 14.98%, 62.1 ± 7.33%, and 37.06 ± 20.0%, respectively, compared to the non-treated biofilm (Fig.3B). Biofilm metabolic activity showed that CP reduced the viability of MDR *E. coli* biofilm cells in a dose-dependent manner, by 85.1 ± 7.9%, 75.9 ± 9.23% and 63.41 ± 1.94%at concentrations of 25, 50, and 100 mg/ml, respectively (Fig. 3C). The CP–AMP combination was effective in inhibiting both preformed biofilm biomass (by 28.96 ± 1.07%) and biofilm viability (by 18.16 ± 6.05%), more than CP or AMP alone. These results demonstrate that the CP–AMP combination was able to both inhibit the formation of biofilm and eliminate existing biofilm of MDR *E. coli*.

### Coffee Pulp–Ampicillin Combination Treatment Disrupts the Morphological Characterization of Multidrug-Resistant *E. coli* and Biofilm Formation

**Fig. 4:**
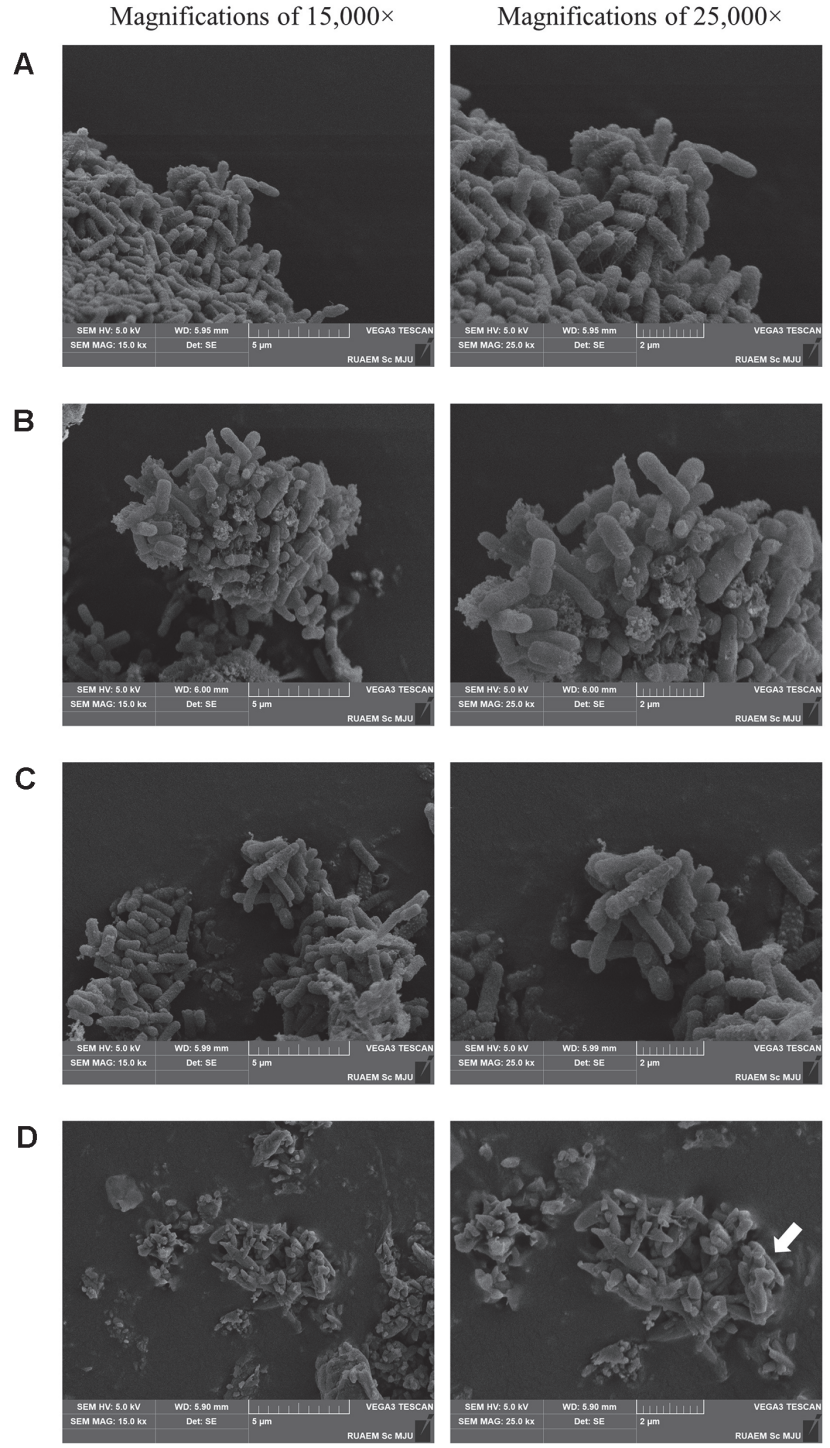
Effects of the coffee pulp–ampicillin (CP-AMP) combination on bacterial cell morphology. The *E. coli* E48 strain was treated for 3 h at 37°C with CP or AMP alone, or the CP–AMP combination. SEM images at 15,000× and 25,000× magnification show: (**A**) the control; (**B**) CP alone at 25 mg/ml; (**C**) AMP alone at 50 mg/mL; and (**D**) the CP–AMP combination treatment (CP 0.2 mg/ml + AMP 0.1 mg/ml). Cell damage is indicated by the arrow.

For a greater understanding of the effect of the CP–AMP combination treatment, we used SEM to investigate the morphology of the cells after treatment for 3 h-bactericidal time-with or without CP and with the CP–AMP combination. Fig. 4 shows SEM images of *E. coli* E48 cells at 15,000× and 25,000× magnification. The non-treated cells had a smooth surface with biofilm formation and an intact cell membrane and no surface ruptures (Fig. 4A), while treatment with CP (Fig. 4B) or AMP (Fig. 4C) alone caused minor membrane damage and decreased biofilm development. Interestingly, bacterial cells were extremely damaged with membrane corrugations, withering, and breaking after treatment with the CP–AMP combination (indicated by the arrow in Fig. 4D).

These results show that the CP–AMP combination impaired cell membrane integrity and eliminated biofilm, resulting in morphological defects that allowed intracellular material leakage, cell membrane shrinking, and, eventually, cell death.

## Discussion

According to a WHO assessment, antimicrobial resistance is one of the top ten global public health problems facing humanity. It is the outcome of drug misuse and overuse, which have reduced antibiotic potency [30]. Therefore, alternative treatment approaches are in high demand. Plant-derived compounds are a well-known source of antibacterial substances [29, 30]. Coffee and tea are the two most popular non-alcoholic beverages worldwide. Green tea (*Camellia sinensis*) extract containing high levels of polyphenol catechins revealed significant antibacterial activity against MDR *E. coli* from clinical specimens of patients implicated in urinary tract infections [31, 32]. Furthermore, the combination of green tea extract and antibiotics has shown a synergistic effect in overcoming antibiotic resistance [33]. Although numerous studies have established anti-*E. coli* activity of natural product extracts, this is the first report of antibacterial activity of coffee beans and coffee-by-product extracts, involving DGC, CP, and AL, against MDR *E. coli* strains. We show that DGC, CP, and AL have anti-MDR *E. coli* properties, and that the CP–AMP combination is a highly effective treatment for MDR *E. coli*, which is resistant to 13 drugs—AMP, AML, KF, CTX, SXT, MEM, IPM, CN, CIP, NOR, AMC, SAM, and TE-by causing membrane permeability disruption and biofilm eradication.

Coffee green beans are unroasted coffee beans used as the raw material for making roasted coffee. Green coffee beans contain many bioactive phytochemicals, including caffeic acid, chlorogenic acid, diterpenes, and trigonelline [34]. Unroasted beans have a higher concentration of some of these compounds, such as chlorogenic acid. In this study, DGC had a high chlorogenic acid content of 12.56 mg/g, followed by caffeic acid of 0.25 mg/g [14]. CP is the outer layer of the coffee berry. After coffee berries are harvested, the pulp is usually discarded as waste. CP contains a variety of bioactive compounds, such as chlorogenic acid, caffeic acid, carotenoids, and phenolic compounds, that have potential health benefits [35]. In a previous study, CP had 13.45 mg/g chlorogenic acid, 1.1 mg/g caffeic acid, and 16.88 mg/g caffeine [14]. Coffee leaves contain several bioactive compounds, some of which have potential health benefits, for example, chlorogenic acid, quinic acid, lignans, and theophylline [36]. It is important to note that AL extracts contain 1.99 mg/g of chlorogenic acid, 0.8 mg/g of caffeic acid, and 17.72 mg/g of caffeine [14]. Flavonoids, chlorogenic acid, caffeic acid, trigonelline, caffeine, and protocatechuic acid play a substantial role as potential natural antibacterial substances against enteric bacteria [37-39]. We recently established that DGC and CP are the most efficient antibacterial agents against clinical MDR *Vibrio cholerae*. Furthermore, the combination of DGC, CP, or AL and TE had a synergistic antibacterial effect on MDR strains [14]. However, the antibacterial activity differed depending on the extract sample and the strain of bacteria. A previous study reported that robusta coffee leaf extract, which includes a high concentration of chlorogenic acid, is effective against foodborne bacteria, such as *S. aureus*, *B. subtilis*, *E. coli*, and *S. typhimurium*, by disrupting bacterial cell membrane integrity [15]. Moreover, the phenotypic and genotypic diversity of coffee plants, the quality of field processing, laboratory extraction processes, and the solvents used are also factors that affect antibacterial activity [36, 40, 41].

The mechanisms of action of the potent bioactive compounds of coffee-chlorogenic acid, caffeic acid, and caffeine - against bacteria are complex and not fully understood, but several studies suggest that they may include the following: (1) disruption of the cell membrane, leading to leakage of cell contents and ultimately cell death [14, 15, 23, 42]; (2) inhibition of the formation of biofilm (bacterial communities that can form on surfaces and can be difficult to eradicate), with some bioactive compounds of coffee, such as caffeine and chlorogenic acid, inhibiting biofilm formation and reducing bacterial adhesion to surfaces [43-46]; and (3) modulation of gene expression, leading to changes in their behavior and bacterial susceptibility to antibiotics [47, 48]. Overall, the mechanisms of action of the bioactive compounds of coffee against bacteria are complex and multifaceted, and further study is needed to fully understand how they work and how they can be exploited to combat bacterial infections.

The development of antibiofilm agents is a potential approach to the management of *E. coli* infections. Mature biofilms are more difficult to treat than early-stage biofilms because they act as a physical barrier against the passage of medications and exhibit greater resistance to treatment. As a result, treating established biofilms can be difficult and may necessitate higher antibiotic doses or alternative treatment options [49, 50]. Our results showed that CP, especially in combination with AMP, inhibits biofilm formation and eradicates mature biofilm. The compounds in CP might inhibit a specific target of biofilm formation, such as the matrix or the signaling mechanisms that allow bacteria to form biofilms. However, the mechanisms need clarification in future studies.

To address the limitations of the present study, future research should focus on investigating the antibacterial activity of the combination treatment against a larger number of MDR *E. coli* strains, including clinical isolates. In addition, further studies should explore the mechanisms of action underlying the effects of the combination therapy, especially against biofilm formation, as well as its efficacy and safety in animal models and eventually in clinical trials.

## Conclusion

We showed that the CP–AMP combination at a concentration of 0.2 mg/ml of CP and 0.1 mg/ml of AMP had synergistic antibacterial activity against MRD *E. coli* strains, more than CP or AMP alone. The combination reduced the MIC of AMP by up to 500-fold (from 50 mg/ml to 0.1 mg/ml). The pharmacokinetics of the CP–AMP combination expressed bactericidal activity within 3 h, and was associated with membrane disruption and biofilm inhibition and eradication. Therefore, the CP–AMP combination in therapeutic regimens is a promising new treatment option for MDR *E. coli* infections.

## Supplemental Materials

Supplementary data for this paper are available on-line only at http://jmb.or.kr.

## Figures and Tables

**Table 1:** The minimum inhibitory concentration (MIC) and minimum bactericidal concentration (MBC) of dried green coffee bean (DGC), coffee pulp (CP), and arabica leaf (AL) extracts (mg/ml) against 28 multidrug-resistant *E. coli* strains.

No.	Strains	DGC		CP		AL	
		MIC	MBC	MIC	MBC	MIC	MBC
1	E1	50	100	12.5	25	50	100
2	E3	50	100	25	50	50	100
3	E5	50	100	25	50	50	100
4	E6	50	100	25	50	50	100
5	E8	50	100	25	50	50	100
6	E9	50	100	25	50	50	100
7	E11	50	100	12.5	25	50	100
8	E14	50	100	25	50	50	100
9	E15	50	100	12.5	25	50	100
10	E16	50	100	25	50	50	100
11	E20	50	100	25	50	50	100
12	E21	50	100	25	50	50	100
13	E24	50	100	25	50	50	100
14	E26	50	100	12.5	25	12.5	25
15	E27	50	100	25	50	50	100
16	E28	50	100	25	50	50	100
17	E30	50	100	25	50	50	100
18	E32	50	100	25	50	12.5	25
19	E34	50	100	25	50	12.5	25
20	E36	50	100	50	100	12.5	25
21	E39	50	100	50	100	50	100
22	E41	50	100	25	50	50	100
23	E48	50	100	25	50	50	100
24	E49	50	100	25	50	50	100
25	E50	50	100	12.5	25	50	100
26	E52	50	100	12.5	25	50	100
27	E65	50	100	12.5	25	50	100
28	E66	50	100	12.5	25	50	100
Reference	ATCC 25922	50	100	25	50	50	100

*MIC and MBC are expressed as mg/ml.

**Table 2:** The susceptibility of the multidrug-resistant *E. coli* E48 strain to dried green coffee bean (DGC), coffee pulp (CP), and arabica leaf (AL) extracts, standard coffee bioactive compounds, and ampicillin (AMP).

Samples	MIC (mg/ml)	MBC (mg/ml)
Crude extracts		
DGC	50	100
CP	25	50
AL	50	100
Coffee bioactive compounds		
Caffeine	6.25	12.5
Chlorogenic acid	12.5	25
Caffeic acid	25	50
AMP	50	100

**Table 3:** The synergistic effect of the coffee pulp–ampicillin (CP–AMP) combination treatment against the multidrug-resistant *E. coli* E48 strain.

Samples	MIC (mg/ml) of extracts [a]		FIC (a)	MIC (mg/ml) of ampicillin [b]		FIC (b)	FICI	Outcome
	Alone	Combination		Alone	Combination			
Crude extracts								
DGC	50	50	1	50	50	1	2	Additive
CP	25	0.2	0.008	50	0.1	0.002	0.01	Synergistic
AL	50	50	1	50	50	1	2	Additive
Coffee bioactive compounds								
Caffeine	6.25	6.25	1	50	50	1	2	Additive
Chlorogenic acid	12.5	12.5	1	50	50	1	2	Additive
Caffeic acid	25	25	1	50	50	1	2	Additive

*FICI ≤0.5: a synergistic effect; FICI >0.5 and ≤4: an additive effect; and FICI >4: an antagonistic effect.
