# Supplementary material for: Restoring Ampicillin Sensitivity in Multidrug-Resistant Escherichia coli Following Treatment in Combination with Coffee Pulp Extracts
Source: J Microbiol Biotechnol. 2023 May 24;33(9):1179–88. doi: 10.4014/jmb.2304.04051 (PMC10580893; doi:10.4014/jmb.2304.04051)
Supplement: Supplementary file 1 [file jmb-33-9-1179-supple.pdf]

J. Microbiol. Biotechnol.
https://doi.org/10.4014/jmb.2304.04051

Supplementary Tables

Table S1. Antimicrobial resistance patterns toward 15 antimicrobial agents isolated from isolated *E. coli* from houseflies in the hospital area.

No.	Isolates	Antimicrobial resistance pattern	Total multi-drug resistant
1	E1	AMP, AML, C, CIP, AMC, SAM, TE	7
2	E3	AMP, AML, KF, CTX, C, SXT, CN, TE	8
3	E5	AMP, AML, KF, CTX, CIP, NOR	6
4	E6	AMP, AML, KF, TE	4
5	E8	AMP, AML, KF, CTX, C, CN, SAM, TE	8
6	E9	AMP, AML, KF, SAM, TE	5
7	E11	AMP, AML, KF, CTX, C, AK, TE	7
8	E14	AMP, AML, KF, AK, TE	5
9	E15	AMP, AML, KF, CTX, C, CN, TE	7
10	E16	AMP, AML, KF, CTX, C, SAM, TE	7
11	E20	AMP, AML, KF, CTX, SXT, SAM	6
12	E21	AMP, AML, KF, C, SXT, CIP, TE	7
13	E24	AMP, AML, KF, CTX, C, SXT, CN, SAM, TE	9
14	E26	AMP, AML, KF, SXT, SAM, TE	6
15	E27	AMP, AML, KF, CTX, SXT	5
16	E28	AMP, AML, KF, CTX, SAM, TE	6
17	E30	AMP, AML, KF, SAM	4
18	E32	AMP, AML, KF, CTX, C, SXT, CN, CIP, SAM, TE	10
19	E34	AMP, AML, KF, SXT, AK, SAM, TE	7

No.	Isolates	Antimicrobial resistance pattern	Total multi-drug resistant
20	E36	AMP, AML, KF, STX, SAM	5
21	E39	AMP, AML, KF, C, SXT, SAM, TE	7

Table S1. (Cont.)

No.	Isolates	Antimicrobial resistance pattern	Total multi-drug resistant
22	E41	AMP, AML, KF, CTX, C, CN, AMC, SAM, TE	9
23	E48	AMP, AML, KF, CTX, SXT, MEM, IPM, CN, CIP, NOR, AMC, SAM, TE	13
24	E49	AMP, AML, KF, CTX, C, SXT, TE	7
25	E50	AMP, AML, KF, C, SXT, CN, SAM, TE	8
26	E52	AMP, AML, SAM, TE	4
27	E65	AMP, AML, KF, CTX, C, SXT, CN, CIP, NOR, AMC, SAM, TE	12
28	E66	AMP, AML, KF, CTX, C, CIP, NOR, AMC, SAM, TE	10

Abbreviations: AMP, Ampicillin; AML, Amoxycillin; KF, Cephalothin; CTX, Cefotaxime; C, Chloramphenicol; SXT, Trimethoprim-sulfamethoxazole; MEM, Meropenem; IMP, Imipenem; AK, Amikacin; CN, Gentamicin; CIP, Ciprofloxacin; NOR, Norfloxacin; AMC, Amoxicillin/clavulanic acid; SAM, Ampicillin/sulbactam; TE, Tetracycline
